# Comparative analysis of obstetric, perinatal, and neurodevelopmental outcomes following chorionic villus sampling and amniocentesis

**DOI:** 10.3389/fmed.2024.1407710

**Published:** 2024-06-28

**Authors:** Nari Kim, Eun Hui Joo, Seoyeon Kim, Taeho Kim, Eun Hee Ahn, Sang Hee Jung, Hyun Mee Ryu, Ji Yeon Lee

**Affiliations:** Department of Obstetrics and Gynecology, CHA Bundang Medical Center, CHA University School of Medicine, Seongnam, Republic of Korea

**Keywords:** chorionic villus sampling, CVS, amniocentesis, cervical length, cerclage, neurodevelopmental outcome

## Abstract

**Background:**

The risks of invasive prenatal tests are reported in previous studies such as miscarriage, fetal anomalies, and bleeding. However, few compare short-term and long-term outcomes between invasive tests. This study aims to investigate obstetric, perinatal, and children’s neurodevelopmental outcomes following chorionic villus sampling (CVS) or amniocentesis in singleton pregnancy.

**Methods:**

This retrospective cohort study included healthy singleton pregnancies underwent transabdominal CVS (gestational age [GA] at 10–13 weeks) or amniocentesis (GA at 15–21 weeks) at a single medical center between 2012 and 2022. Only cases with normal genetic results were eligible. Short-term and long-term neurodevelopmental outcomes were evaluated.

**Results:**

The study included 200 CVS cases and 498 amniocentesis cases. No significant differences were found in body mass index, parities, previous preterm birth, conception method, and cervical length (CL) before an invasive test between the groups. Rates of preterm labor, preterm premature rupture of the membranes, preterm birth, neonatal survival, neonatal short-term morbidities, and long-term neurodevelopmental delay were similar. However, the CVS group had a higher rate of cervical cerclage due to short CL before 24 weeks (7.0%) compared to the amniocentesis group (2.4%). CVS markedly increased the risk of cervical cerclage due to short CL (adjusted odd ratio [aOR] = 3.17, 95%CI [1.23–8.12], *p* = 0.016), after considering maternal characteristics.

**Conclusion:**

Performing CVS resulted in a higher incidence of cerclage due to short cervix or cervical dilatation compared to amniocentesis in singleton pregnancies. This highlights the importance of cautious selection for CVS and the necessity of informing women about the associated risks beforehand.

## Introduction

Invasive prenatal diagnostic tests such as chorionic villus sampling (CVS) and amniocentesis are necessary to confirm genetic abnormalities in the fetus during pregnancy (1). Although non-invasive prenatal testing (NIPT) is advancing, its ability to detect genetic mutations is still limited, so invasive prenatal tests such as CVS or amniocentesis are required to confirm the diagnosis. Invasive prenatal testing remains an essential diagnostic tool (1–3).

CVS is usually conducted between 10 and 13 weeks of gestation, involving the aspiration of placental villi using a needle or specialized catheter under ultrasound guidance. Amniocentesis, typically performed between 15 and 20 weeks or later, retrieves amniotic fluid using a needle under ultrasound guidance (4). Extensive research has examined procedure-related risks, such as miscarriage and fetal anomalies, following each prenatal diagnostic test (5–7).

Previous systematic reviews and meta-analyses have shown low or negligible procedure-related risks of miscarriage compared to similar chromosomal abnormality backgrounds (6, 8). However, limited research has focused on obstetric outcomes and the short-and long-term prognosis of newborns following amniocentesis and CVS for prenatal diagnosis.

Thus, we conducted this study to investigate obstetric, perinatal, and children’s long-term neurodevelopmental outcomes following prenatal invasive testing, particularly CVS or amniocentesis, in singleton pregnancies.

## Methods

In this retrospective cohort study, we examined singleton pregnant women who underwent either CVS or amniocentesis for prenatal diagnosis and subsequently delivered at our hospital between January 2012 and December 2022. Approval for this study was obtained from the Institutional Review Board at CHA Bundang Medical Center (IRB No. 2023-11-034-003). We excluded cases involving multiple gestations, structural fetal abnormalities, abnormal chromosomal results from CVS or amniocentesis, and pregnancies with unknown outcomes. Pregnant women were categorized into two groups based on the type of invasive prenatal test: (1) CVS and (2) amniocentesis. These procedures were performed when women presented an elevated risk of fetal genetic disorder, indicated by factors such as advanced maternal age, abnormal maternal serum markers, structural fetal abnormalities identified by ultrasonography including increased nuchal translucency thickness, previous history of fetal chromosomal anomalies, or parental genetic disorders.

CVS procedures were conducted between 10 and 13 weeks under sterile conditions using a double-needle technique. This involved inserting an 18-gage needle as a trocar, followed by a smaller 20-gage needle into the placenta. Negative pressure was created with a 20-cc syringe, and the needle was moved up and down through the placenta several times while maintaining the negative pressure. Amniocentesis was performed between 15 and 21 weeks of gestation. A small-gage needle, often 21-or 22-gage, was placed into the amniotic sac, with care to avoid the fetus, umbilical cord, and placenta when possible. The first few milliliters of fluid were discarded to avoid maternal contamination, and typically, 20–30 mL of amniotic fluid was collected. Pregnant women who underwent CVS or amniocentesis at our medical center routinely attended follow-up consultations 2 to 3 weeks after the procedures. During these visits, ultrasound examinations were conducted to assess the status of the fetus and uterus.

We assessed obstetric and perinatal measures as short-term outcomes in neonates born to women who had undergone CVS or amniocentesis. Short-term outcomes included pulmonary hypertension, transient tachypnea of the newborn (TTN), respiratory distress syndrome (RDS), bronchopulmonary dysplasia (BPD), patent ductus arteriosus (PDA), intracranial hemorrhage (ICH), retinopathy of prematurity (ROP), meconium aspiration syndrome (MAS), and necrotizing enterocolitis (NEC).

We employed a similar approach to assess children’s neurodevelopment as in our previous study (9). Long-term neurodevelopmental outcomes were evaluated after 1 year of corrected age. Developmental delay was diagnosed if babies did not reach expected milestones, assessed using Bayley-III tests and/or the Gross Motor Function Measure. In cases where neurodevelopmental outcomes were not tested, developmental status was assessed by reviewing academic performance records within the medical records. A child was considered to have no developmental delay if they demonstrated an achievement equivalent to a score of 20% or higher in the academic achievement evaluation for reading, writing, arithmetic, and related subjects conducted by the Korea Institute for Curriculum and Evaluation.

We utilized the Chi-square test for categorical variables and the Student *t*-test for continuous variables in our analysis. Multivariate analysis was conducted with maternal age and body mass index (BMI) as covariates. Statistical significance was set at *p* < 0.05. All analyses were conducted using SPSS software (version 28.0, SPSS Institute, Chicago, IL, United States).

## Results

After applying exclusion criteria, a total of 200 CVS and 498 amniocentesis cases were included from 1,048 singleton pregnant women. Baseline clinical characteristics were analyzed (Table 1). Maternal age was significantly higher in the amniocentesis group (36.5 ± 4.2) than in the CVS group (35.2 ± 4.7, *p* = 0.030). However, BMI, parity, history of preterm birth, and method of conception showed no significant differences. CVS and amniocentesis were performed at mean gestational ages of 11.8 weeks and 16.8 weeks, respectively (*p* = 0.05). The mean cervical length (CL) at the time of prenatal invasive testing was 4.2 cm for the CVS group and 3.9 cm for the amniocentesis group (*p* = 0.814).

**Table 1:** Clinical characteristics of the study population.

	CVS (*N* = 200)	Amniocentesis (*N* = 496)	*p*-value
Maternal age (year)	35.2 ± 4.7	36.5 ± 4.2	0.030
BMI in pre-pregnancy	23.3 ± 4.5	22.8 ± 3.7	0.522
Nulliparity	189 (94.5)	466 (93.6)	0.396
Prior preterm birth	2 (1.0)	6 (1.2)	0.586
Method of conception			0.073
Spontaneous	116 (58.0)	199 (40.1)	
Ovarian stimulation	14 (7.0)	22 (4.5)	
*In vitro* fertilization	70 (35.0)	275 (55.4)	
GA at the time of the invasive test (weeks)	11.8 ± 0.7	16.8 ± 1.4	0.005
Cervical length at the time of the invasive test (cm)	4.2 ± 0.9	3.9 ± 0.9	0.814

Data given as mean ± SD or number of cases (percentage); CVS, chorionic villus sampling; BMI, body mass index; GA, gestational age.

The analysis of obstetric outcomes (Table 2) revealed no significant differences in rates of preeclampsia, placenta previa, or antenatal admission due to preterm labor after 20 weeks gestation compared to the amniocentesis group. However, the rate of cerclage after prenatal invasive testing was significantly higher in the CVS group after adjusting for maternal age and BMI (adjusted odds ratio [aOR] = 4.50, 95%CI [1.09–9.48], *p* = 0.01). The incidence of cerclage due to short cervix or cervical dilatation showed a significant difference, with similar gestational ages at cerclage between the two groups. The CVS group exhibited a markedly higher incidence of cerclage due to short cervix or cervical dilatation compared to the amniocentesis group (7.0% vs. 2.4%, aOR = 3.17, 95%CI [1.23–8.12], *p* = 0.016). Additionally, the incidence of gestational diabetes mellitus (GDM) was lower in the CVS group compared to the amniocentesis group (11.1% vs. 16.9%, aOR = 0.46, 95%CI [0.22–0.94], *p* = 0.034).

**Table 2:** Obstetric outcomes.

	CVS (*N* = 200)	Amniocentesis (*N* = 496)	*p*-value	Adjusted odds ratio (95% CI)^a^ (Reference: Amniocentesis)	*p*-value
Preeclampsia	6 (3.0)	20 (4.0)	0.529	0.87 (0.49–2.17)	0.293
Gestational diabetes mellitus	22 (11.1)	84 (16.9)	0.033	0.46 (0.22–0.94)	0.034
Placenta previa	3 (1.5)	22 (4.4)	0.062	1.04 (0.73–3.03)	0.678
Antenatal admission due to preterm labor after GA 20 weeks	29 (14.6)	91 (18.3)	0.243	0.95 (0.53–1.70)	0.868
Cerclage	28 (14.1)	32 (6.4)	0.001	4.50 (1.09–9.48)	0.005
Cerclage due to short cervix or cervical dilatation	14 (7.0)	12 (2.4)	0.004	3.17 (1.23–8.12)	0.016
GA at the time of cerclage (weeks)	18.5 ± 3.9	18.8 ± 3.8	0.430		

Data given as mean ± SD or number of cases (percentage); CVS, chorionic villus sampling; GA, gestational age.

a All outcomes were adjusted for maternal age and body mass index.

We assessed the short-term and long-term outcomes of newborns (Table 3). There were no differences in GA at birth, birthweight, small for gestational age, APGAR score at 5 min <7, or neonatal intensive care unit (NICU) admission between the two groups. Furthermore, short-term neonatal morbidities during hospitalization, including ICH, ROP, MAS, neonatal jaundice, TTN, RDS, BPD, pulmonary hypertension, and NEC showed no significant difference between the two groups. The risk of developmental delay was assessed as a long-term outcome, with no significant difference between CVS and amniocentesis groups after adjusting for maternal age and BMI.

**Table 3:** Postnatal short-term and long-term outcomes.

	CVS (*N* = 200)	Amniocentesis (*N* = 496)	*p*-value	Adjusted odds Ratio (95% CI)^a^ (Reference: Amniocentesis)	*p*-value
GA at delivery (weeks)	38.4 ± 2.2	38.1 ± 2.0	0.842		
**Preterm birth**					
23^+0^–27^+6^ weeks	2 (1.0)	2 (0.4)	0.324	1.81 (0.16–20.48)	0.631
23^+0^–31^+6^ weeks	4 (2.0)	8 (1.6)	0.750	1.41 (0.26–7.56)	0.691
23^+0^–33^+6^ weeks	7 (3.5)	19 (3.8)	0.842	1.13 (0.35–3.63)	0.844
23^+0^–36^+6^ weeks	21 (10.5)	67 (13.5)	0.288	0.84 (0.42–1.70)	0.629
Cesarean delivery	106 (53.0)	292 (58.9)	0.092	0.97 (0.62–1.51)	0.879
Birth weight (gram)	3158.3 ± 538.4	3054.4 ± 550.4	0.569		
SGA	17 (2.1)	6 (2.1)	0.947	0.60 (0.26–1.40)	0.240
Apgar score at 5 min < 7	4 (2.0)	8 (1.6)	0.718	0.90 (0.10–8.47)	0.928
NICU admission	26 (13.0)	85 (17.1)	0.184	0.60 (0.31–1.15)	0.123
NICU hospitalization(days)	22.2 ± 30.5	25.1 ± 30.2	0.647		
**Morbidity during hospitalization**	**(*N* = 198)**	**(*N* = 495)**			
Sepsis	11 (5.5)	23 (4.6)	0.625	1.29 (0.45–3.69)	0.636
Meconium aspiration syndrome	7 (3.5)	15 (3.0)	0.739	1.11 (0.35–3.52)	0.858
Neonatal jaundice	42 (21.0)	110 (22.1)	0.753	0.91 (0.52–1.57)	0.721
Transient tachypnea of newborn	5 (2.5)	14 (2.8)	0.819	0.94 (0.26–3.47)	0.927
Respiratory distress syndrome	8 (4.0)	33 (6.6)	0.182	0.41 (0.12–1.40)	0.155
Bronchopulmonary dysplasia	2 (1.0)	6 (1.2)	1.000	0.76 (0.09–6.58)	0.799
Pulmonary hypertension	1 (0.5)	1 (0.2)	0.491	3.14 (0.19–51.02)	0.421
Composite morbidity^b^	55 (27.5)	141 (28.3)	0.829	0.80 (0.48–1.34)	0.402
**Long-term outcomes**					
Developmental delay	9/198 (4.5)	25/495 (5.1)	0.781	0.95 (0.34–2.66)	0.929

Data given as mean ± SD or number of cases (percentage); CVS, chorionic villus sampling; GA, gestational age; SGA, small for gestational age; NICU, neonatal intensive care unit.

a All outcomes were adjusted for maternal age and body mass index.

b Composite morbidity during hospitalization includes neonatal sepsis, intracerebral hemorrhage, retinopathy of prematurity, meconium aspiration syndrome, neonatal jaundice, transient tachypnea of newborn, respiratory distress syndrome, bronchopulmonary dysplasia, pulmonary hypertension and necrotizing enterocolitis.

## Discussion

To our knowledge, this is the first study to compare obstetric and postnatal outcomes, including long-term development, between CVS and amniocentesis in singleton pregnancies. Our findings underscore a higher incidence of cerclage due to short cervix or cervical dilatation in the CVS group compared to the amniocentesis group.

Fetal membranes, comprising the amnion and chorion/placenta, play crucial roles in fetal protection, maintaining pregnancy, and initiating labor. They undergo remodeling at both cellular and matrix levels throughout gestation to accommodate the growing intrauterine volume. Matrix metalloproteinase-mediated extracellular matrix degradation, which is involved in inflammatory processes, orchestrates this process (10). During prenatal invasive tests performed under sterile conditions, infection from the procedure would minimally affect the sterile amniotic fluid unless an inflammation source from the mother or fetus is identified beforehand (11–13). However, the CVS and amniocentesis procedures themselves can irritate the fetal membranes and cause stress on the chorion and/or amniotic membrane.

The fetal membrane stays in a sterile condition. Fetal membrane, with or without infection, can lead to adverse outcomes during pregnancy such as spontaneous preterm labor, preterm premature rupture of membrane, and cervical insufficiency (14–16). The fetal membrane is susceptible to inflammatory conditions with or without the detection of microorganisms, leading to adverse obstetric and neonatal outcomes (17–20). Several studies noted the sterile inflammation, known as inflammation without detecting microorganisms, of fetal membranes in asymptomatic patients with a sonographic short cervix (14). Multiple studies have linked a sonographic short cervix in the mid-trimester with chorioamniotic inflammation, increasing the risk of adverse pregnancy outcomes (21–23). Sterile inflammation’s impact on complications like preeclampsia and preterm labor during pregnancy is extensively documented in the literature (24, 25).

Although research on sterile inflammation, particularly triggered by mechanical stress, is limited, Nadue-Vallee et al. suggested that this process can be stimulated via tissue injury or cell death through sterile pathways in reproduction and pregnancy (24). Recent investigations have explored fetal membrane inflammation induced by clinical insults like hypoxia and oxidative stress, offering comprehensive evidence of sterile inflammatory substances such as damage-associated molecular patterns (DAMPs) released from the fetal membrane (26). Stress-induced damage to the fetal membrane leads to irreversible cell cycle alterations, resembling the environment observed in infectious inflammation (27, 28). This triggers tissue damage pathways, potentially leading to preterm birth, alongside non-infectious risk factors such as mitogen-activated protein kinase (MAPK) activation, cellular senescence, and antibody-mediated immune responses (20). During CVS, fetal membranes receive greater physical stimulation than amniocentesis, as the needle diameter is larger and a wider area of the fetal membrane is stimulated. Additionally, CVS is performed earlier in pregnancy than amniocentesis, exposing the fetal membranes to inflammatory conditions for a longer duration. Therefore, it is hypothesized that the number of cervical cerclage due to short cervix or cervical dilatation was higher in the CVS group than in the amniocentesis group in this study. However, our study showed no significant difference in the gestational age at delivery, rate of preterm birth, and perinatal outcomes in short-or long-term between the CVS and amniocentesis cases. We speculate that early detection of the short cervix by sonography or physical examination within 2–4 weeks after CVS or amniocentesis and performing cerclage may have contributed to preventing preterm birth and subsequent adverse outcomes for newborns.

In our study, we observed a higher incidence of GDM in the amniocentesis group compared to the CVS group. GDM influences maternal blood levels of human β-chorionic gonadotropin (hCG), alpha-fetoprotein (AFP), unconjugated estriol (uE3), and pregnancy-associated plasma protein-A (PAPP-A), which are maternal serum screening markers. This can affect the false positive or negative rates of screening tests for autosomal trisomies (29, 30). Reports frequently indicate reduced levels of first-trimester β-hCG in diabetic women (31–33). Raty et al. found significant differences in maternal serum β-hCG and AFP levels between pregnant women with GDM and controls (34). Hur et al. identified uE3 and β-hCG as predictors of GDM development in early pregnancy (35). In addition, reduced levels of first-trimester PAPP-A were inversely related to hemoglobin A1C, reflecting glycemic control (36–38). Thus, the higher incidence of GDM in cases where amniocentesis was performed may not be due to amniocentesis itself but rather to amniocentesis being conducted in women with abnormal results of the maternal serum screening, some of whom already had undiagnosed GDM or were likely to develop it.

This study has several strengths. To the best of our knowledge, it is the first to comprehensively examine obstetric, perinatal, and children’s neurodevelopmental outcomes following CVS and amniocentesis in singleton pregnancies. Additionally, it exclusively focuses on singleton pregnancies with thorough follow-up at a single medical center, enhancing the consistency of the data. However, the study has some limitations. Sterile inflammatory cytokines were not confirmed, and there were variations in indications for CVS and amniocentesis, as well as inconsistent assessment tools for developmental delays. These limitations underscore the necessity for further prospective studies to generalize these findings. Future research should involve interdisciplinary collaboration among specialists, including obstetricians, neonatologists, and pediatricians.

## Conclusion

There were no notable differences in obstetric and short-and long-term newborn outcomes between CVS and amniocentesis. However, CVS was associated with an increased risk of short CL before 24 weeks, leading to a higher likelihood of subsequent cerclage. This insight emphasizes the importance of cautious candidates’ selection for CVS, considering the potential risk of subsequent short CL requiring cerclage. Additionally, it underscores the necessity of informing women about the risk in advance.

## Data availability statement

The raw data supporting the conclusions of this article will be made available by the authors, without undue reservation.

## Ethics statement

The studies involving humans were approved by Institutional Review Board at CHA Bundang Medical Center. The studies were conducted in accordance with the local legislation and institutional requirements. Written informed consent for participation was not required from the participants or the participants’ legal guardians/next of kin in accordance with the national legislation and institutional requirements.

## Author contributions

NK: Conceptualization, Data curation, Investigation, Methodology, Software, Visualization, Writing – original draft. EJ: Data curation, Formal analysis, Investigation, Methodology, Project administration, Visualization, Writing – original draft. SK: Data curation, Formal analysis, Investigation, Validation, Writing – review & editing. TK: Formal analysis, Methodology, Software, Visualization, Writing – review & editing. EA: Investigation, Resources, Validation, Writing – original draft, Writing – review & editing. SJ: Data curation, Methodology, Project administration, Software, Validation, Writing – original draft, Writing – review & editing. HR: Funding acquisition, Investigation, Supervision, Validation, Writing – review & editing. JL: Conceptualization, Data curation, Formal analysis, Investigation, Methodology, Project administration, Resources, Software, Supervision, Visualization, Writing – original draft, Writing – review & editing.
